# Design Improvement of Four-Strand Continuous-Casting Tundish Using Physical and Numerical Simulation

**DOI:** 10.3390/ma16020849

**Published:** 2023-01-15

**Authors:** Quanhui Li, Bangming Qin, Jiangshan Zhang, Hongbiao Dong, Ming Li, Biao Tao, Xinping Mao, Qing Liu

**Affiliations:** 1 State Key Laboratory of Advanced Metallurgy, University of Science and Technology Beijing, Beijing 100083, China; 2 Nanjing Iron & Steel United Co., Ltd., Nanjing 210035, China; 3 School of Engineering, University of Leicester, Leicester LE1 7RH, UK; 4 Collaborative Innovation Center of Steel Technology, University of Science and Technology Beijing, Beijing 100083, China

**Keywords:** tundish metallurgy, flow pattern, numerical simulation, physical simulation

## Abstract

The flow pattern is vital for the metallurgical performance of continuous casting tundishes. The purpose of this study was to design and optimize the flow characteristics inside a four-strand tundish. Numerical simulations and water model experiments were validated and utilized to investigate the flow behavior. The effect of different flow rates in the original tundish was evaluated; two modified retaining walls and a new ladle shroud were designed for optimization. The molten steel inside the original tundish tends to be more active as the flow rate increases from 3.8 L/min to 6.2 L/min, which results in a reduction in dead volume from 36.47% to 17.59% and better consistency between different outlets. The dead volume and outlet consistency inside the tundish are improved significantly when the modified walls are applied. The proper design of the diversion hole further enhances the plug volume from 6.39% to 13.44% of the tundish by forming an upstream circular flow in the casting zone. In addition, the new trumpet ladle shroud demonstrates an advantage in increasing the response time from 152.5 s to 167.5 s and alleviating the turbulence in the pouring zone, which is beneficial for clean steel production.

## 1. Introduction

Continuous casting is the fundamental process of modern metallurgy, and it plays a crucial role in solidifying molten steel into quality blanks [1,2,3]. During the continuous casting process, tundish serves as a reservoir between the ladle and the mold, and it was initially intended to divert the steel and ensure the continuity of steel production. In recent years, the tundish has developed more metallurgical functions as the quality of steel has improved, such as removing inclusions and adjusting the temperature field [4,5,6,7,8,9,10], which are significantly influenced by the flow field inside the tundish. There is usually a temperature gradient in the tundish, and a 7–14 K difference is reported in Ref. [9], which can be narrowed by various techniques, such as induction heating [10]. In order to optimize the metallurgical behavior of the tundish, the flow pattern of the tundish has been frequently investigated.

Numerical simulation and hydraulic modelling are the most common research methods since the melt inside the tundish cannot be directly observed. The cost of experiments could be reduced significantly by numerical simulation [5], and the accuracy of numerical results can be verified [11,12]. With a focus on the control of the flow field in tundish, some kinds of flow control devices have been investigated to achieve desirable performance [13,14,15,16,17], such as turbulence inhibitors, dams, weirs, and walls. Heaslip et al. [18] studied the influence of turbulence inhibitors on the flow inside tundish and found that their use increases the negative effect of eddy near the fluid jet. The dams in a multistrand tundish were examined by Delgado-Ramirez et al. [19], and the results showed that the proposed dam design can improve the flow pattern and removal of inclusions. Recently, the ladle shrouds were also reported to function as a flow control device by adjusting the incoming jet from the ladle [20], which demonstrates benefits in various tundishes [21,22,23]. The aforementioned studies have contributed significantly to the understanding of different transport processes in tundish operations, which has led to a more efficient design of tundishes. However, few studies have been conducted to optimize the tundish by combining the design of retaining walls and ladle shrouds.

The current study aimed to investigate the flow pattern inside a four-strand tundish. Numerical simulations and water model experiments were carried out to qualitatively evaluate the flow inside the tundish. The flow patterns of the original tundish at different flow rates were first compared. Then two modified designs of retaining walls were provided for optimization, and their residence time distribution curve (RTD curves) and flow field were analyzed. Moreover, to further improve the metallurgical performance of the tundish, a new ladle shroud was also proposed.

## 2. Materials and Methods

Numerical simulation and physical simulation are both significant ways to investigate the tundish metallurgy. The results obtained by numerical simulation can be verified by the physical model to ensure the accuracy of the study. The modelling conditions, theories, settings, and validation results are discussed in the following subsections.

### 2.1. Geometry and Meshing

**Figure 1:**
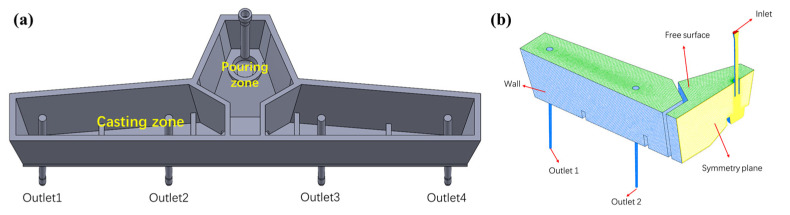
Geometry and mesh of the studied models: (**a**) four-strand tundish; (**b**) mesh.

**Figure 2:**
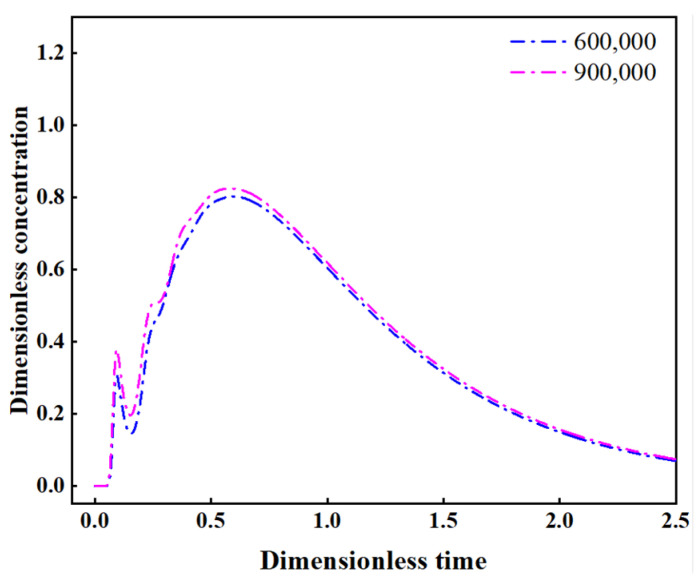
Comparison of RTD curves under different mesh sizes.

The subject of this study is a four-strand tundish operating in a Chinese steelmaking plant; its geometric dimensions are illustrated in Figure 1a. As presented in Figure 1b, half of the tundish was constructed as the computational domain, which could save computing resources and reduce the calculation time; the time consumption to converge to 10e-4 decreases from 3353 s to 1467 s to reach a steady flow field in the current case. To determine an appropriate number of grids, three densities of mesh (around 300,000, 600,000, and 900,000) were divided for comparison. The results show that the coarsest mesh (300,000) cannot achieve convergence. The RTD curves obtained by 600,000 and 900,000 cells show no significant difference, as illustrated in Figure 2. Therefore, the whole domain was divided into about 600,000 polyhedral cells for simulation.

### 2.2. Design of Flow Control Devices

**Figure 3:**
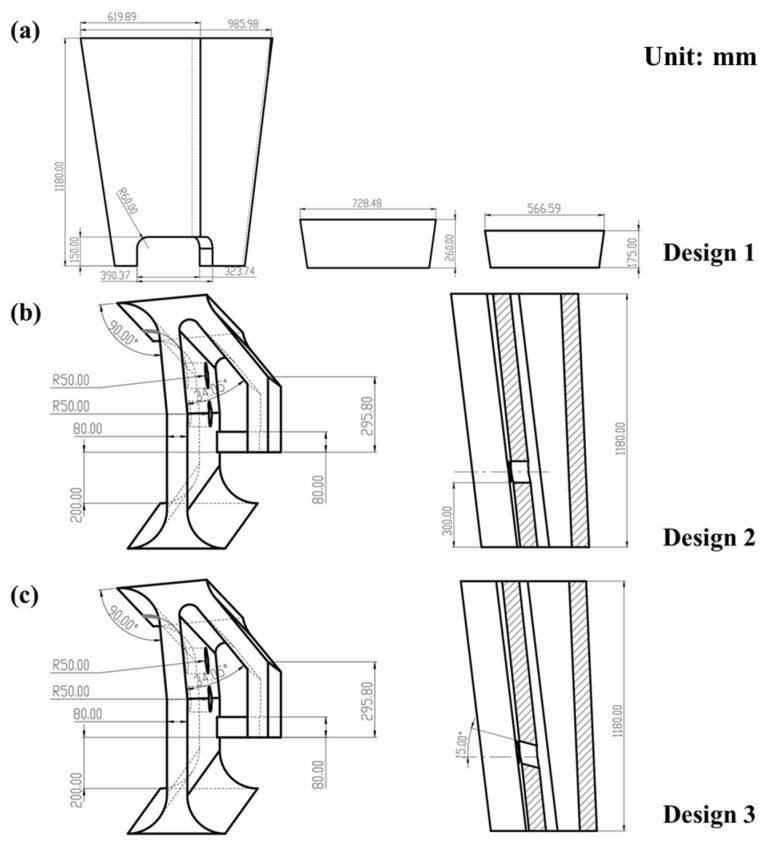
Schematics of the flow control devices: (**a**) Design 1; (**b**) Design 2; (**c**) Design 3.

Three different designs of retaining walls were adopted to compare the flow inside the tundish. Figure 3 shows the detailed dimensions of these devices. Design 1 is the structure used in the actual production, which contains walls and dams. A rectangular hole is located at the bottom of the retaining wall, as shown in Figure 3a, and two dams with a thickness of 80 mm are set at a distance of 460 mm and 1908 mm from the retaining wall. Design 2 and Design 3 are the optimized structures that consist of a wall with two diversion holes in it. The diversion holes have the potential to form a large circular flow in the casting zone and activate the flow around the far outlets, which brings about Design 2. As Figure 3b shows, two diversion holes with 100 mm diameters are located at a height of 300 mm from the bottom of the tundish. In addition, the angle of the diversion hole plays a crucial role in forming an optimal flow field, and a proper angle could be beneficial for prolonging the residence time of the incoming flow and allows more opportunity for inclusion removal in the casting zone. The angle of the diversion hole was then adjusted, which brings about Design 3. Two holes with upward angles of 15° are set at the same position as Design 2, and the diameters of the holes are also 100 mm. The specific parameters of the model are shown in Figure 3 and Table 1.

**Figure 4:**
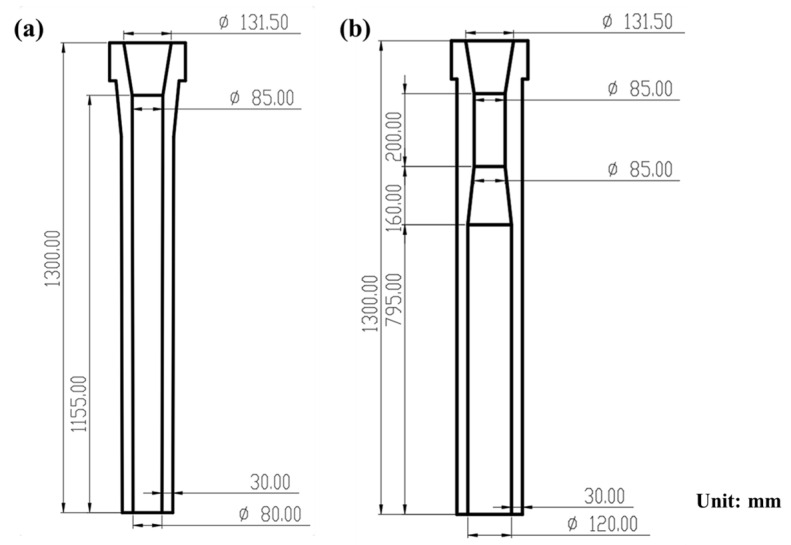
The geometry of the tested two ladle shrouds: (**a**) CLS; (**b**) TLS.

Moreover, a new trumpet ladle shroud was designed, as shown in Figure 4. The first one is the conventional ladle shroud (CLS) that is currently utilized, which consists of a tube with a relatively small inner diameter (ϕ80 mm). The other is the trumpet-shaped ladle shroud (TLS), which is divided into a divergent chamber and a straight tube with a larger inner diameter (ϕ120 mm). The TLS was designed mainly based on our previous studies [23,24]. The basic design rules are that the length of the trumpet section should be at least four-fold of the inner diameter of the shroud outlet; the outlet size should not be larger than the inner diameter of the turbulence inhibitor.

### 2.3. Mathematical Modelling

The basic equations describing the flow of steel include the continuity equation, momentum equation, and k-ε equations. The species transport model was used to calculate the residence time of the melt. These equations are given as follows:

1. Continuity equation:

(1) $$\frac{\partial \rho }{\partial t}+\nabla \cdot \left(\rho \vec{v}\right)=0$$

2. Momentum equations:

(2) $$\frac{\partial \left(\rho \vec{\nu }\right)}{\partial t}+\nabla \cdot \left(\rho \vec{\nu }\vec{\nu }\right)=-\nabla p+\nabla \left|\mu_{\mathrm{eff}}\left(\nabla \cdot \vec{\nu }\right)\right|+\rho g+\vec{F}$$

where *ρ* is the density of the fluid, kg/m^3^; $\vec{\nu }$ is the velocity, m/s; *p* is the pressure, Pa; $\mu_{\mathrm{eff}}$ is the effective viscosity; and $\vec{F}$ is the source term of the additional force, N/m^3^.

3. The turbulence is defined by the standard k-ε model as follows:

(3) $$\frac{\partial \left(\rho k\right)}{\partial t}+\begin{array}{c} \frac{\partial \left(\rho v_{i}k\right)}{\partial x_{i}}=\frac{\partial }{\partial x_{j}}\left|\left(\mu +\frac{\mu_{t}}{\sigma_{k}}\right)\frac{\partial k}{\partial x_{j}}\right|+G_{k}-\rho \varepsilon \end{array}$$

(4) $$\begin{array}{c} \frac{\partial \left(\rho \varepsilon \right)}{\partial t}+\rho \frac{\partial \left(\rho \varepsilon v_{i}\right)}{\partial x_{i}}=\frac{\partial }{\partial x_{j}}\left|\left(\mu +\frac{\mu_{t}}{\sigma_{k}}\right)\frac{\partial \varepsilon }{\partial x_{j}}\right|+C_{1\varepsilon }\frac{\varepsilon }{k}G_{k}+C_{2\varepsilon }\frac{\varepsilon^{2}}{k} \end{array}\rho$$

where the $G_{k}$ and $\mu_{t}$ is given by:

(5) $$G_{k}=\mu_{t}\frac{\partial v_{j}}{\partial x_{i}}\left(\frac{\partial \nu_{i}}{\partial v_{j}}+\frac{\partial v_{j}}{\partial v_{i}}\right)$$

(6) $$\begin{array}{c} \mu_{t}=\rho C_{u}\frac{k^{2}}{\varepsilon } \end{array}$$

where the constant values of *C*_1ε_, *C*_2ε_, *C_μ_*, *σ*_k_, and *σ*_ε_ are 1.44, 1.92, 0.09, 1.0, and 1.3, respectively [25].

4. The diffusion behavior of the tracer can be described by the conservation equation, which is expressed as follows:

(7) $$\frac{\partial }{\partial t}\left(\rho C\right)+\frac{\partial \left(\rho v_{i}C\right)}{\partial x_{i}}=\frac{\partial }{\partial x_{i}}\left(\rho D_{\mathrm{eff}}\frac{\partial C}{\partial x_{i}}\right)$$

where *C* is the concentration of the tracer and $D_{\mathrm{eff}}$ is the effective diffusivity.

The boundary conditions of the mathematical model are given below.

1. A velocity inlet was applied to the inlet of the tundish. The turbulent kinetic energy and turbulent dissipation rate were calculated by *k* = 0.01*u*^2^_inlet_ and ε = 2*k*^1.5^/*d*_inlet_.
2. The outlet of this model was defined as an outflow boundary.
3. The free surface of the steel bath is flat and frictionless.
4. In the symmetric plane, the gradients of all variables were set to zero.
5. The walls of the tundish were treated using the non-slip wall boundary condition as well as the near-wall surface using the standard wall function.

ANSYS FLUENT was used to simulate the fluid flow in the tundish, and the following assumptions were adopted:

1. The molten steel in the tundish is an incompressible single-phase turbulent flow.
2. The influence of the slag layer on the flow is ignored.
3. Both water and tracers are assumed as liquid phases.

The standard k-ε model was applied as the turbulence model, which is widely used in the simulation of the steel-making process, and its accuracy has been verified in many cases [26,27,28]. The pressure-based solver was selected, and the SIMPLEC algorithm was used to couple pressure and velocity. The calculation converged when the residuals were less than 10^−4^. Moreover, the steady flow field was first obtained when calculating the RTD curve or the mixing behavior of ink. The species transfer model was then employed, and the tracer was added at the inlet of the tundish. RTD curves were calculated by monitoring the tracer concentration at each outlet of the tundish. Detailed information regarding the properties of steel and other parameters for calculation is provided in Table 1.

### 2.4. Validation between Mathematical and Water Modelling

**Figure 5:**
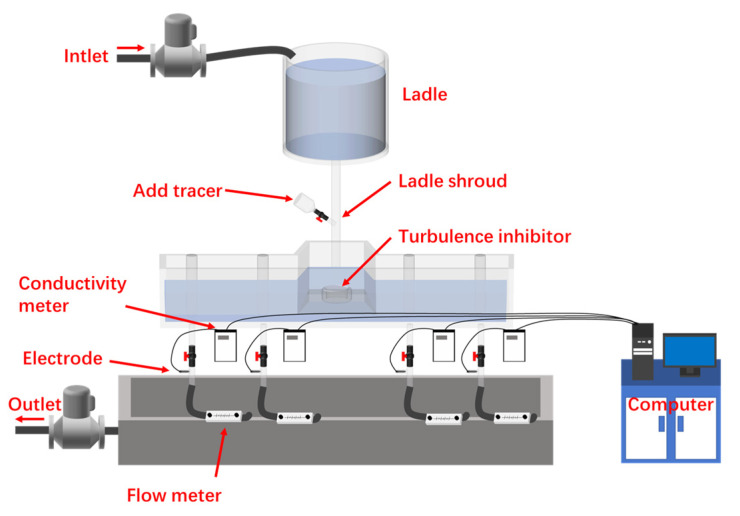
The *λ* = 1:3 physical model of the actual tundish.

This study was carried out on a *λ* = 1:3 scaled-down physical model of the actual tundish, which can be seen in Figure 5. Similitude theory was used to determine the specific parameters for the water model experiments, mainly including geometric and dynamic similarities. Froude numbers (Fr = *v*^2^/*gL*) were ensured to be equal between the physical and numerical simulation. In addition, the Reynolds number between the actual and the scaled-down model was calculated at the entrance by:

(8) Re=ρvdμ   

where *ρ* is the density of fluid, *v* is the velocity of fluid, *d* indicates the entrance diameter of the shroud, and *μ* is the dynamic viscosity. The Reynolds number of the actual and the scaled-down model is 55,835 and 9093, respectively, both in the range of turbulence.

To verify the results between the water model experiments and simulations, the RTD curves and the dispersion of ink inside the tundish of Design 2 were obtained. KCl (potassium chloride) saturated solution was considered as the tracer. The flow rate at each outlet was set at 4.7 L/min and 50 mL of KCl solution was added into the tundish when the flow reached a stable state. The electrical conductivity at each outlet was monitored by the electrode for 1650 s. The obtained conductivity was then converted to a dimensionless concentration in order to calculate the RTD curve. Furthermore, the experiments were repeated at least twice to ensure the accuracy of the results.

The RTD curves represent one of the most widely used methods for quantitative analysis of the flow field and consistency between streams in multi-strand tundish [29,30], and the residence time of the melt is an important factor when evaluating metallurgical improvements in tundish based on different turbulence control devices.

The average dimensionless concentration $\bar{C}$ of individual strand *i* (*i* = 4) can be expressed as:

(9) $$\begin{array}{c} \bar{C}=\frac{1}{n}\overset{n}{\underset{i=1}{\sum }}C_{i} \end{array}$$

The mean residence time $\bar{t}$ can be expressed by the following equations:

(10) $$\bar{t}=\frac{\int_{0}^{\infty }\bar{c}tdt}{\int_{0}^{\infty }\bar{c}dt}$$

The dead volume fraction is calculated by:

(11) $$V_{d}=1-\frac{\bar{t}}{t_{0}}$$

where $t_{0}$ means the theoretical residence time, which is considered as:

(12) $$\begin{array}{c} t_{0}=\frac{V_{\mathrm{model}-\mathrm{tundish}}}{V_{\mathrm{outflow}-\mathrm{rate}}} \end{array}$$

where $V_{\mathrm{model}-\mathrm{tundish}}$ is the volume of the fluid inside model and $V_{\mathrm{outflow}-\mathrm{rate}}$ is the total outflow rate.

The plug volume fraction is expressed by:

(13) $$V_{p}=\frac{t_{\min}}{t_{0}}$$

Therefore, the well-mixed volume can be obtained as follows:

(14) $$V_{m}=1-V_{d}-V_{p}$$

The consistency is defined by the overall standard deviation of RTD curves, which can be calculated by:

(15) $$S=\frac{1}{N}\overset{N}{\underset{j=1}{\sum }}\sqrt{\frac{\sum_{i=1}^{n}{\left[E_{i}\left(\theta_{j}\right)-E\left(\theta_{j}\right)\right]}^{2}}{n}}$$

where *N* is the amount of measured data; *E*_*i*_(*θ*_*j*_) is the dimensionless concentration of the *i*th stream at moment *j*; *E*(*θ*_*j*_) is the average dimensionless concentration at moment *j*; and *n* is the number of strands.

**Figure 6:**
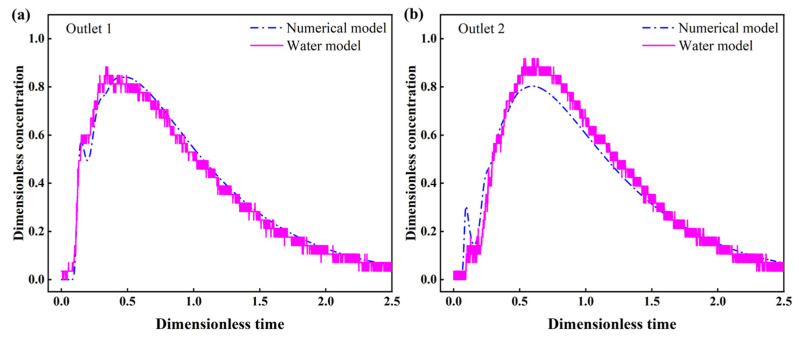
Comparison of RTD curves between numerical and water modelling results: (**a**) outlet 1; (**b**) outlet 2.

Since the tundish is assumed to be symmetrical and halved (1350 mL KCl solution), the RTD curves at the first and second outlets can be used to depict the entire tundish. A comparison between the mathematical and water modelling RTD curves is shown in Figure 6. It is shown that the simulated RTD curves for the first and second outlets demonstrate good consistency with those obtained from water modelling.

**Figure 7:**
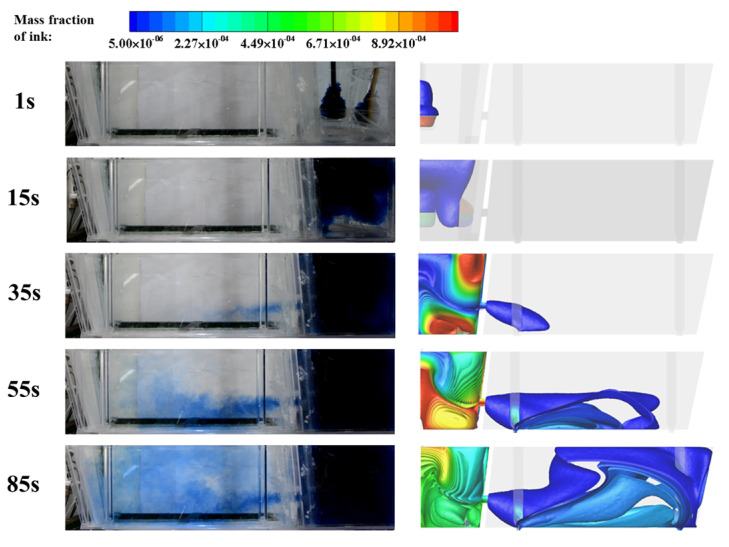
Comparison of ink dispersion between numerical and water modelling results at different times.

Ink diffusion experiments were also performed to further verify the numerical results. The experimental conditions were the same as the RTD experiment except that the KCl was replaced by ink as the tracer. Videos and snapshots were taken to observe the morphology of ink at different times. As shown in Figure 7, the comparison of ink dispersion profiles at different times shows good agreement, which further indicates the reliability of the modelling methods.

Compared with physical modelling, numerical simulations could save costs considerably, and the parameters of different designs can be easily adjusted. Thus, the following study was mainly conducted by numerical simulation.

## 3. Results and Discussion

The flow inside the tundish is affected by various factors, including the flow rate, structure of the tundish, design of the retaining wall and ladle shroud, etc., which will be discussed in the following subsections.

### 3.1. The Effect of Flow Rate on the Flow Pattern of the Original Tundish

**Figure 8:**
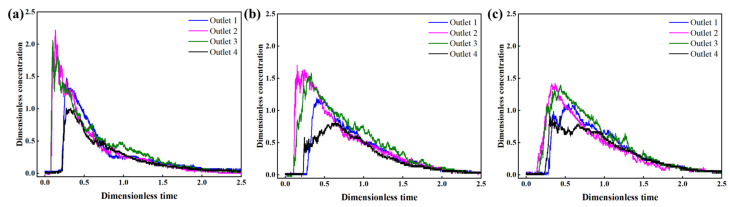
The RTD curves in different outlets under three flow rates: (**a**) 3.8 L/min; (**b**) 4.7 L/min; (**c**) 6.2 L/min.

The flow inside the original tundish with different flow rates was investigated. To establish the actual process comprehensively, three typical flow rates at each outlet were considered based on the cast speed, which are 3.8 L/min, 4.7 L/min, and 6.2 L/min in the water model experiments. Figure 8 shows the RTD curves obtained from the outlets under different flow rates, and apparent differences can be seen. The maximum dimensionless concentration at the near outlets (outlet 2 and outlet 3) reached more than 2.0 under the flow rate of 3.8 L/min, which is much higher than that of the far outlets (outlet 1 and outlet 4). This indicates that there is a short circuit flow when the flow rate is low, which could cause the uneven mixing of the molten steel and be detrimental to provide sufficient time for the removal of inclusions [31]. It is also seen that the peak concentration decreases to 1.5 or lower when the flow rate increases to 4.7 L/min and 6.2 L/min, and the four curves from different outlets tend to be more similar, which implies better consistency between the outlets.

**Figure 9:**
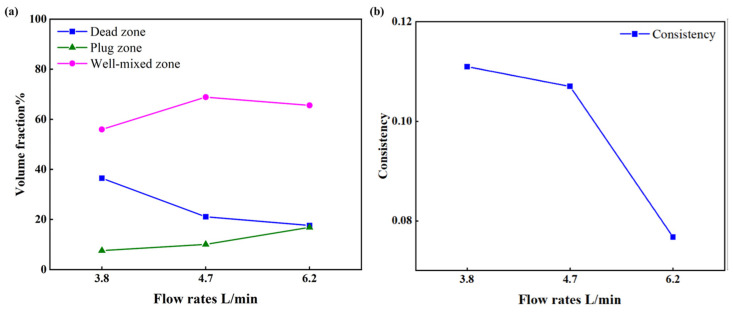
The volume fraction of flow and the consistency of RTD curves under different flow rates: (**a**) Volume fraction of flow; (**b**) Consistency of RTD curves.

To quantitatively assess the effect of flow rates on the flow pattern inside the tundish, the volume fraction of the flow and consistency of RTD curves under different flow rates are compared in Figure 9. As illustrated in Figure 9a, the volume rate of the dead zone decreases from 36.47% (3.8 L/min) to 21.07% (4.7 L/min) and 17.59% (6.2 L/min), respectively, which means the dead volume of 6.2 L/min is 51.77% smaller than that of 3.8 L/min. The volume rate of the plug volume increases from 7.64% (3.8 L/min) to 10.07% (4.7 L/min) and 14.67% (6.2 L/min). Moreover, the volume rates of the well-mixed volume are 55.89% (3.8 L/min), 68.85% (4.7 L/min), and 67.74% (6.2 L/min), respectively. These results suggest that the dead volume inside the tundish becomes smaller and the plug volume becomes larger with the increase in flow rate, while the well-mixed volume shows no significant correlation with the flow rate. In addition, the consistency between different outlets under three flow rates is presented in Figure 9b. It is noted that the consistency under lower flow rates was poor, which may lead to an uneven temperature distribution and cleanliness, and relatively higher flow rates could be beneficial to narrow the difference between the four outlets. A possible explanation for this relationship might be that the fluid inside the original tundish, especially in the casting zone, tends to be more active at higher flow rates. These findings indicate the advantage of higher flow rates in alleviating poor consistency and improving the flow pattern inside the origin tundish. However, these results are inconsistent with the conclusions obtained in some publications, such as the work by He et al. [32], which shows that the increase in flow brings about a higher dead volume due to the reduction in the mean residence time, as traditionally believed. As for this four-strand tundish, the increased flow rates activate the melt motion around the far outlets, which narrows the difference between the near and far strands; as a result, the dead volume is reduced by the raised flow rates. Thus, the effect of flow rate is structure-dependent on the change in dead volume.

### 3.2. The Fluid Flow Inside the Tundish under Three Designs of Retaining Wall

**Figure 10:**
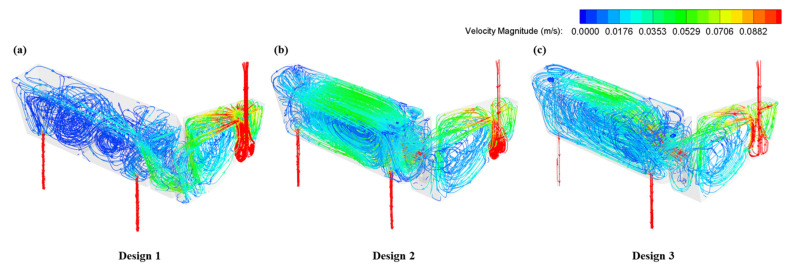
The velocity trajectory inside tundish under different designs of wall: (**a**) Design 1; (**b**) Design 2; (**c**) Design 3.

The design of the retaining wall is the key to adjusting the flow pattern inside the casting zone of the tundish pool. Herein, three designs, including the original tundish (Design 1), were compared at a flow rate of 4.7 L/min. As shown in Figure 10, the molten steel first flows into the pouring zone of the tundish through the ladle shroud, where the turbulence inhibitor is impacted by the high-speed fluid. The stream then distributes into the tundish and the velocity profile tends to be stable. There is no remarkable difference observed between the three designs in the pouring zone of the tundish. However, differences emerge when the fluid reaches the pouring zone. The molten steel goes through the hole placed at the bottom of the wall and encounters the dam in Design 1, which will form an upward stream around the near outlet. Moreover, with the development of the stream, the momentum of fluid becomes weak, and some small circular eddies are generated around the far outlet due to the dam. It is apparent that the velocity profile inside the Design 1 tundish is uneven, which may deteriorate the consistency of different outlets.

**Figure 11:**
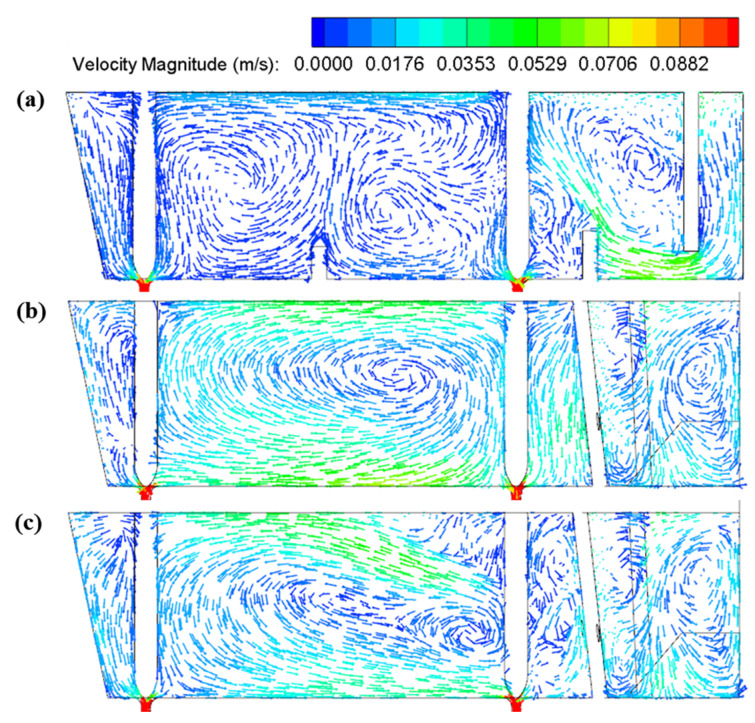
The velocity vectors at the longitudinal section through outlets: (**a**) Design 1; (**b**) Design 2; (**c**) Design 3.

The retaining wall with diversion holes has the potential to form a large circular flow in the casting zone and activate the flow around the far outlets. In the case of Design 2, a large circular flow is developed since the molten steel passes across the diversion hole at the retaining wall. The velocity vectors at the longitudinal section through outlets are presented in Figure 11. A clockwise circular flow is formed due to the downward dragging forces from the outlets as seen in Figure 11b; this circular flow distributes the momentum of fluid elements more evenly inside the pouring zone, which could effectively improve the mixing behavior and narrow the difference between outlets. However, the clockwise flow is not beneficial for prolonging the residence time of incoming flow and allows less opportunity for inclusions removal in the casting zone. In the case of design 3, the angle of diversion is adjusted to form an upward degree of 15°, as illustrated in Figure 3c. It is seen that a large counterclockwise circular flow is formed inside the pouring zone thanks to the upward angle of the diversion hole. As shown in Figure 11c, the counterclockwise flow pattern first makes the molten steel flow to the free surface of the tundish after flowing through the diversion hole. This flow pattern could not only improve the uniformity of molten steel (such as temperature) but also prolong the contact time between the steel and slag, which can increase the probability of the inclusion removal.

**Figure 12:**
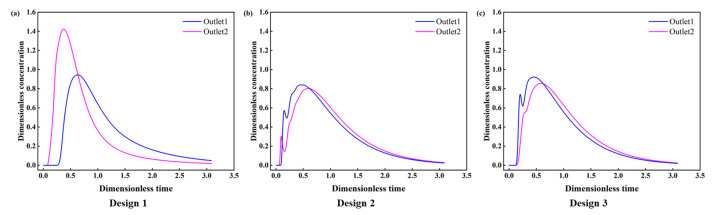
The RTD curves for different designs of retaining wall: (**a**) Design 1; (**b**) Design 2; (**c**) Design 3.

To further understand the effect of the retaining wall on the flow pattern in the tundish, RTD curves of the three designs of the wall were calculated as shown in Figure 12. It can be seen that the maximum dimensionless concentration of Design 1 is much higher than that of Design 2 and Design 3, which may be attributed to the short-circuit flow inside the tundish. Table 2 provides the response time and the volume fraction of flow using the three wall designs. The volume rate of the dead zone decreases from 20.88% (Design 1) to 14.53% (Design 2) and 14.27% (Design 3) gradually, which could be attributed to the relatively higher velocity in the pouring zone of Design 2 and Design 3, as mentioned above. The volume rate of the plug volume is 8.64% (Design 1), 6.39% (Design 2), and 13.44% (Design 3); correspondingly, the response time is 98 s (Design 1), 72.5 s (Design 2), and 152.5 s (Design 3), respectively, which indicates that the response time could be effectively prolonged by the counterclockwise circular flow in Design 3. Furthermore, the consistency of different outlets also improves in the case of Design 2 and Design 3; the variance decreases from 0.108 (Design 1) to 0.025 (Design 3) and 0.027 (Design 3). This may benefit from the circular flow in the pouring zone, which could mix the steel bath evenly. These results indicate the significant advantages of Design 3 in improving the flow pattern of the tundish, which will promote the mixing and uniformity of the steel bath.

### 3.3. Effect of Different Ladle Shrouds on the Flow Field of the Tundish

In order to further optimize the tundish, a new trumpet ladle shroud is designed and examined under the structure of Design 3.

**Figure 13:**
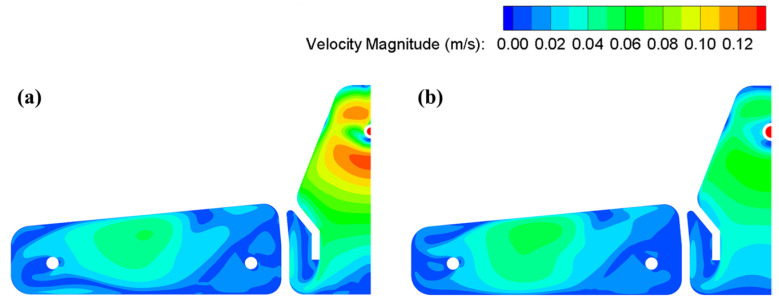
Surface velocity contours of the tundish under (**a**) CLS and (**b**) TLS.

Figure 13 illustrates the surface velocity contours of the tundish under different ladle shrouds. As previously described, liquid steel has a higher velocity in the pouring zone, and the surface velocity maximizes at around 0.12 m/s. The TLS tends to calm the surface velocity of the steel bath in the pouring zone and the maximum velocity is halved to about 0.06 m/s. This is due to the fact that the trumpet design of the TLS alleviates the turbulence of the jet into the pouring zone [33], and the surface velocity is effectively lowered, which is beneficial to reduce the risk of melt exposure to the atmosphere and related contamination and heat loss [34,35].

**Figure 14:**
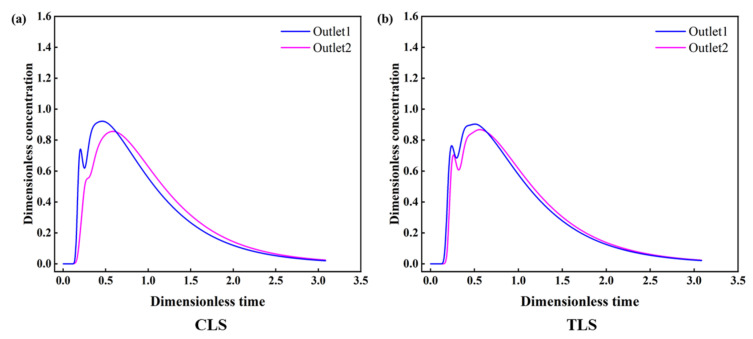
The RTD curves obtained using different ladle shrouds: (**a**) CLS; (**b**) TLS.

A comparison of the RTD curves with different ladle shrouds was also made. As shown in Figure 14, no significant variations were observed in the morphology of the RTD curves. Table 3 provides the response times and the volume fraction using different ladle shrouds. It can be seen that the volume rate of the plug volume increases slightly from 13.44% (CLS) to 14.76% (TLS). Additionally, the response time has been extended from 152.5 s (CLS) to 167.5 s (TLS), which could benefit the possibility of inclusion removal. Another benefit of TLS is that the consistency index of the far and near outlets is improved, which may be caused by better pre-mixing of the tracer in the pouring volume due to the prolonged response time. Thus, a combination of the optimized retaining wall and TLS is suggested for this four-strand tundish for better metallurgical performance, which has the potential to enhance the flow pattern and outlet consistency. For actual application, the new retaining wall can be used without difficulty. As the TLS features a larger volume, its weight is a little heavier than that of CLS, and practice is required to realize the immersion ladle opening to maximize its performance for clean steel production.

## 4. Conclusions

Both numerical and physical models have been established to optimize the flow pattern inside a four-strand tundish, and their results match well. The conclusions of this study are summarized as follows:

1. The molten steel inside the original tundish pool tends to be more active, especially the zone around the far outlets, when the flow rate is increased from 3.8 L/min to 4.7 L/min and 6.2 L/min, which results in a decrease in dead volume and a better consistency between different outlets.
2. The dead volume is relatively high and the consistency between the near and far outlets remains to be improved in the original tundish. Two optimized flow control devices (Design 2 and Design 3) have been proposed. A large circular flow is formed in Designs 2 and Design 3, which contributes to the decrease in dead volume and the improvement of consistency.
3. Design 3 outperforms Design 2 in the flow pattern, dead volume, plug volume, and outlet consistency due to the counterclockwise circular flow formed by the upward diversion hole.
4. A new ladle shroud was designed, which further improved the flow characteristics, especially in relieving the turbulence around the impact zone, slightly increasing the plug volume and outlet consistency. Overall, a combination of Design 3 and TLS demonstrates the best performance in the flow characteristics.

## Figures and Tables

**Table 1:** Geometrical parameters and fluid properties.

Parameter	Value
Total length of the shroud, mm	1300
Diameter of the entrance nozzle, mm	80
Inlet diameter of the ladle shroud, mm	131.5
Height of the liquid bath, mm	1000
Width of the liquid bath, mm	2100
Length of the liquid bath, mm	7240
Submergence depth of ladle shroud, mm	300
Liquid steel density, kg·m^−3^	7020
Steel viscosity, kg/(m·s)	0.006
Cross section, mm	420 × 320
Cast speed, m/min	0.4~0.65
Tracer molecule diffusion coefficient, m^2^·s^−1^	1.994 × 10^−9^
Ink molecule diffusion coefficient, m^2^·s^−1^	2.818 × 10^−10^

**Table 2:** Response time and the volume fraction under different wall designs.

Type	Outlet	Volume Rate of the Dead Volume—%	Volume Rate of the Plug Volume—%	Response Time—S	Volume Rate of the Well-Mixed Volume—%	Consistency
Design 1	1st	4.84	27.94	317	67.22	0.108
	2nd	35.34	8.24	93.5	56.42	
	Whole	20.88	8.64	98	70.48	
Design 2	1st	17.87	9.17	104	72.97	0.025
	2nd	11.14	6.13	69.5	82.72	
	Whole	14.53	6.39	72.5	79.08	
Design 3	1st	18.21	13.13	149	68.66	0.027
	2nd	10.26	14.76	167.5	74.7	
	Whole	14.27	13.44	152.5	72.29	

**Table 3:** Response time and volume fraction using different ladle shrouds.

Type	Outlet	Volume Rate of the Dead Volume—%	Volume Rate of the Plug Volume—%	Response Time—S	Volume Rate of the Well-Mixed Volume—%	Consistency
CLS	1st	18.21	13.13	149	68.66	0.027
	2nd	10.26	14.76	167.5	74.7	
	Whole	14.27	13.44	152.5	72.29	
TLS	1st	16.16	14.23	161.5	69.61	0.014
	2nd	12.08	16.79	190.5	71.13	
	Whole	14.13	14.76	167.5	71.11	

## Data Availability

Not applicable.
